# Student-Generated Questions as Opportunities for Sensemaking in Undergraduate Biology

**DOI:** 10.1093/iob/obag039

**Published:** 2026-07-15

**Authors:** S Dabholkar, S Benjamin, Y Wang, J Svoboda

**Affiliations:** Indian Institute of Science Education and Research, Pune, Maharashtra 411008, India; Bunker Hill Community College, Boston, MA 02129, USA; Mount Holyoke College, South Hadley, MA 01075, USA; Tufts University, Medford, MA 02155, USA

## Abstract

Research in science education highlights the value of sensemaking—the process of identifying gaps in understanding and constructing explanations to fill them—for student learning and engagement. Yet college students often perceive undergraduate biology classrooms as prioritizing correct answers over their own questions and ideas. In this paper, we argue that eliciting students’ questions about what they find confusing can create opportunities for them to engage in creative and critical thinking to make sense of biological phenomena. We illustrate this conceptual argument with examples drawn from three interviews with college students about evolutionary biology. We show how framing interviews around students' questions led to productive biological sensemaking and conclude by discussing implications for how biology instructors might foster a sensemaking approach in their classrooms.

## Introduction

*I often find myself being really confused like this, especially in bio …. But if I keep thinking about it, and they don’t even ask me about it, it’s like maybe I should just memorize what’s quizzed.*

*—Layla*

 

*[T]he test won’t ask me about the part that I’m confused about…, which is why I just ignore it.*

*—Nikita*

 

*I think focusing on the right answer limits my brainstorming, you know what I mean? That’s why I was- I originally chose [to talk about] what I know was right.*

*—Will*

The above statements were made by college students reflecting on their learning experiences in introductory biology courses. They represent a pattern evident in both the biology education research literature (Lin et al. 2015; Gouvea et al. 2019; Google et al. 2023) and our own experiences as educators—that students often perceive biology courses, particularly at the introductory level, as valuing answers over thinking and questioning. This perception has implications for how students approach learning in biology (Google et al. 2023). As illustrated in the quotes above, students understand that assignments and exams will value answers, and that the correctness of those answers, not the quality or complexity of students’ thinking, is what will earn them points. It makes sense then to employ more surface-level approaches to learning than to spend time trying to deeply understand the things that they are confused about (Elby 1999; Yerdelen-Damar & Elby 2016; Google et al. 2023). For us, and we suspect for many biology instructors, these student perceptions run counter to our learning goals. We want students to identify and grapple with their own questions to build understandings that make sense to them rather than to recall knowledge for the purpose of reproducing answers (Berland et al. 2016). Beyond that, we want students to recognize that exploring their own questions and confusions is a valuable practice, one that can sustain their engagement with science and support their identities as science learners (Danielak et al. 2014).

Our purpose in this paper is to highlight the value of supporting opportunities for *sensemaking* in biology education. Broadly, the term sensemaking in science education describes learning in which students actively “figure things out” for themselves as opposed to being told (Bierema et al. 2017; Kapon 2017; Southard et al. 2017; Schwarz et al. 2017, 2021; Zhao et al. 2021; Adams & Barth-Cohen 2024). We draw specifically on Odden and Russ’s (2019a) definition of sensemaking as “a process of constructing and revising putative explanations in order to resolve a gap or inconsistency in one’s understanding” (p. 5). According to this definition, sensemaking comprises two interrelated processes. First, sensemaking involves identifying gaps, inconsistencies, or contradictions in one’s understanding of a phenomenon. This aspect of sensemaking aligns with what Phillips and colleagues (2018) have called “problematizing.” As these authors elaborate, noticing, articulating, and convincing others that gaps or contradictions are problematic requires intellectual effort and helps motivate the need to build new knowledge (ibid). Second, sensemaking entails working to construct or revise knowledge to address those gaps or contradictions. This aspect of sensemaking captures the creative, generative thinking required to propose possible explanations, models, or hypotheses (e.g., Southard et al. 2017).

We find Odden and Russ’s (2019a, b) definition of sensemaking useful because it explicitly values both the construction of knowledge and the recognition of what does not yet make sense. The latter—the process of generating the questions that can motivate sensemaking—has received less attention in the literature and is a focus of this paper. We show how framing interviews as about learning more about what students identify as puzzling or contradictory in biology can support sensemaking. To illustrate our argument, we present excerpts from three student interviews about natural selection. We use these interviews to examine when and why students shift from concerns about correctness into sensemaking. Before introducing these examples, we first ground our work in the theoretical construct of framing, which describes the processes through which people make sense of what is appropriate and expected of them in different situations (Tannen & Wallet 1987).

### The role of framing in supporting sensemaking

Scholarship in science education shows that, under the right conditions, students across a range of ages can engage in sensemaking (e.g., Manz 2015; Bierema et al. 2017; Kapon 2017; Bolger et al. 2021). Crucially, students must experience something as genuinely in need of “figuring out” and feel that their attempts to do so will be valued (Berland & Reiser 2011; Manz 2015; Schwarz et al. 2017). While framing generally refers to how people interpret situations, *epistemological* framing refers to how students perceive what counts as knowledge and what role they play in producing it within a given setting (Hammer et al. 2005; Elby & Hammer 2010). Research suggests that many typical school settings convey to students that knowledge consists of established facts and that their job is to recall and report them (Jiménez-Aleixandre et al. 2000; Schwarz et al. 2025). This epistemological framing is reflected in the student quotes at the opening of this paper, in which students described their understanding that biology class is a place for answers, not questions or ideas. Shifting this framing can help students feel comfortable sharing their own ideas and questions and be willing to attempt constructing their own tentative explanations.

Curricular designs that support sensemaking therefore must encourage epistemological framings that signal to students that there is value in constructing knowledge, not just recalling it. Such curricula often include activities that present students with things that don’t immediately make sense (Manz 2015; Bolger et al. 2021; Svoboda & Hayes 2026). For example, Bierema and colleagues (2017) describe an activity in which students are told that a mutation in a gene causes uncontrolled cell growth and are asked to generate a model that could explain this. Such activities invite students to actively construct and evaluate knowledge rather than learn the authoritative explanation—in line with the Odden and Russ (2019a) definition of sensemaking.

Sensemaking activities work best when students are genuinely attempting to construct an explanation that makes sense to them rather than focusing on an answer that they think will please an instructor. As Odden and Russ (2019b) argue, when students generate their own questions about what doesn’t make sense to them, they are more likely to engage in and persist in sensemaking efforts. Curricula designed to support students in problematizing for themselves present complex phenomena without prescribing which specific gaps or contradictions students will take up (Bolger et al. 2021; Manz 2015; Phillips et al. 2018). Students are invited not only to construct knowledge but also to decide which things they want to better understand. Research shows not only that students can generate scientific questions and problems but also that they find these experiences engaging and meaningful, reporting that posing and investigating their own questions supports their emerging identities as scientists (Bolger et al. 2021, Svoboda & Hayes 2026).

In this conceptual paper, we elaborate on the concept of sensemaking by examining when and why sensemaking is likely to emerge for college biology learners. We present empirical analyses of interviews with college students to illustrate how interview framing shaped the emergence of students' questions and ideas, and why this makes framing a valuable lever for supporting sensemaking in biology education.

### Sensemaking in interviews about natural selection

We draw on data from a study of college students’ reasoning about evolution. As part of that research, we interviewed students recruited from first-semester introductory biology courses that are focused on cells and molecules—one course taught at a community college and one at a private university. We chose to recruit students from this type of course because we wanted to elicit their reasoning about evolution by natural selection rather than have them report to us what they were learning in class. Of course, college-level students are not completely new to learning about natural selection; many students reported having been taught about it in high school. And though the classes were focused primarily on cells and molecules, they did include review of the basic ideas of evolution by natural selection. Still, we hoped to uncover ideas and questions that might remain for these students. While studying sensemaking was not the initial focus of this research, we noticed a common pattern across many interviews. Interviews began with a focus on reporting ideas and concepts students had remembered about evolution from prior classroom instruction. Later the interviews shifted, with students posing and reasoning about their own questions. Once we noticed this pattern, subsequent interviews were designed more intentionally to elicit a shift into sensemaking. We examined interviews designed to elicit sensemaking in this paper.

**Fig. 1:**
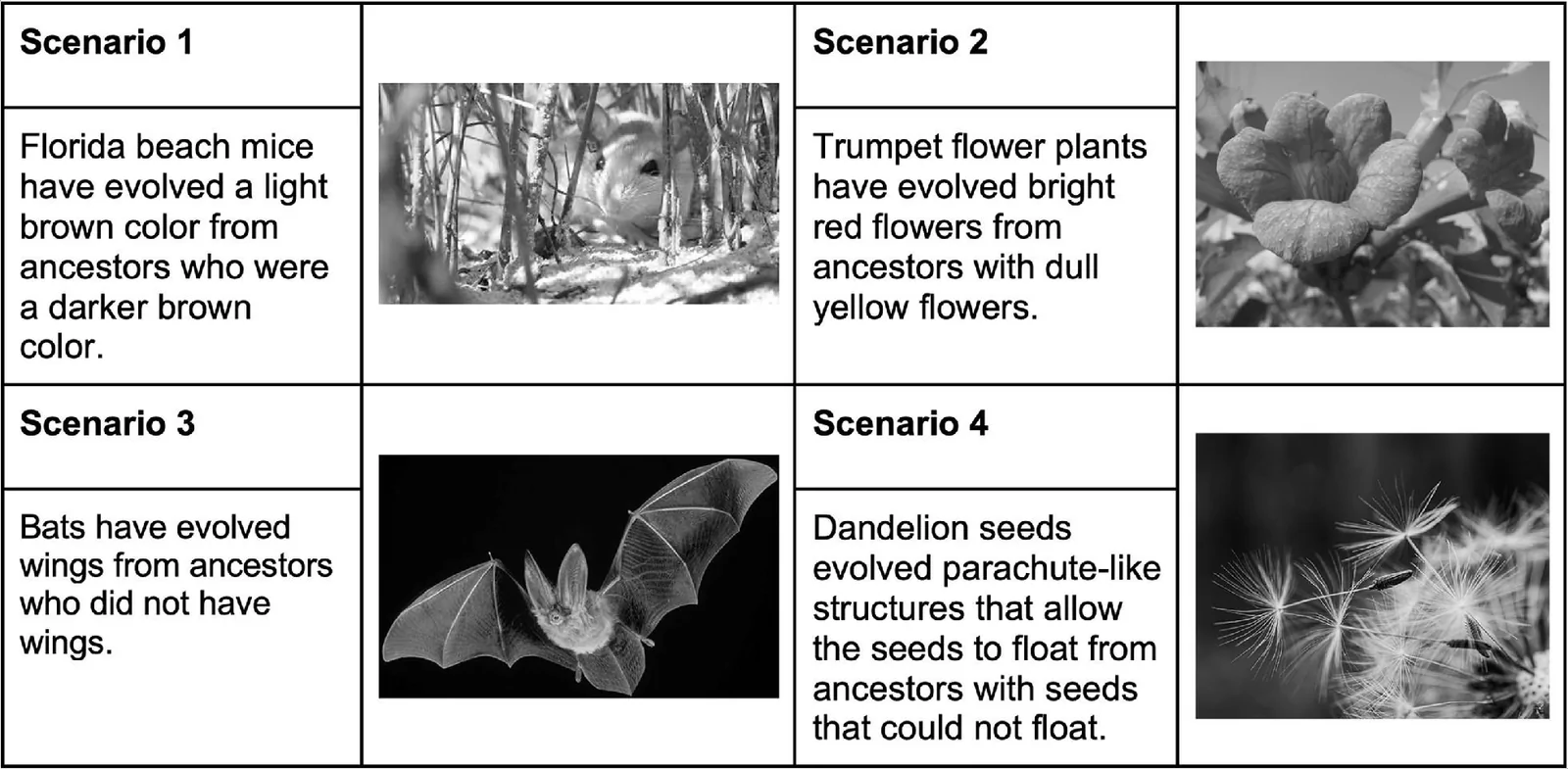
Students were presented with four scenarios and asked to “Pick a scenario from the above and explain how the new trait evolved from ancestors that did not have the trait.” Images (top left) Florida Beach Mouse, Florida Department of Environmental Protection, Public domain, via Wikimedia Commons (top right) Trumpet flower, Audrey from Central Pennsylvania, USA, CC BY 2.0 via Wikimedia Commons (bottom left) Bat, PD-USGov, exact author unknown, Public domain, via Wikimedia Commons (bottom right) Dandelion seeds, Oleksandr K, CC0, via Wikimedia Commons.

While all interviews exhibited evidence of sensemaking, we have chosen to share excerpts from our analysis of three interviews for this paper. These were chosen because they typify the broader set while also illustrating variation in the aspects of natural selection that motivated each student’s sensemaking—making them “information-rich” cases (Merriam 1988). The three interviews were conducted with students who were enrolled in an introductory biology course at a community college taught by the same instructor. The interviewer started each interview by asking students about their interests to help them feel more at ease and by broadly framing the interview as about the students’ “thinking.” The interviewer then presented each student with their written answers to a question that they had answered in class and asked them to reflect on their response. The question was introduced in class as an assignment intended to get students to engage in “critical thinking” and that it would be graded for completion rather than correctness. The question asked students to choose from among four organisms (bat, dandelion, mouse, trumpet flower) and write out an explanation for the evolution of a trait (wings, parachute seeds, fur color, flower color) (Fig. 1). Specifically, students were asked to explain how the trait could have evolved from an ancestral population that did not have that trait. After students were asked to reflect on their written answers the interviewer began to try to elicit sensemaking.

In analyzing these cases, we drew on the work of Russ and colleagues to describe evidence of two possible framings guiding students’ behavior during interviews (2012). The first, a *reporting knowledge* framing describes students’ focus on conveying correct knowledge to the interviewer. Empirical markers of this framing include steady speech and gaze, minimal pausing, and use of formal vocabulary. Students operating within a reporting knowledge framing tend to become apologetic when uncertain, expressing concern over whether their answers would be deemed correct by an authority figure. The second, a *sensemaking* framing, describes moments when students appear to understand the interview as a collaborative exploration of ideas. In this framing, confusion, uncertainty, and partial or tentative thinking are more acceptable. Empirical markers include a combination of shifting gaze, frequent pausing, hedging language (e.g.,, “maybe,” “I think”), and more informal vocabulary. Inferences about framings were generated through collaborative discussion and multiple viewings of interviews among the authors.

In the next sections, we first describe when and why students shifted from reporting knowledge to sensemaking during interviews. We then present three examples of students' sensemaking about evolutionary phenomena. These examples showcase the specific knowledge gaps or questions that students identified as well as their attempts to generate novel, tentative explanations. We end by discussing what these interviews suggest about how to support sensemaking in biology classrooms.

#### When and why do students engage in sensemaking?

We began this paper with quotes from the three focal students explaining why they *do not* engage in sensemaking in introductory biology courses: when expected to produce correct answers, they withhold both their confusions and ideas. And yet, even in answer-focused contexts, hints of students' ideas and wonderings are visible. The written explanations that Layla, Nikita, and Will provided to explain the evolution of different traits (dandelion seeds, bat wings, and flower color, respectively) contain both expected core ideas as well as hints of their questions and ideas (Table 1). All three students incorporated the core ideas of natural selection: differential success, genetic variation, and inheritance (Nehm and Reilly 2007; Gregory 2009; Nehm et al. 2012). Each response also hints at unresolved questions and tentative ideas.

**Table 1:** Students’ written responses to an in-class question shown in Fig. 1.

Layla	I think the dandelion seeds evolved parachute-like structures that allow them to float so that the seeds could disperse to greater distances. These great distances allow dandelions to grow in and adapt to different environments where they can survive better, further from competition. [The trait came from] some type of genetic mutation. After one dandelion experiences this mutation, the offspring will probably inherit the same trait. Reproduction of dandelions with parachute-like structures allow for the structure to become the most common.
Nikita	Bats have evolved wings from ancestors who did not have wings through natural selection. There may have been a gradual change in their environment where flying would be an advantage for the bat’s survival so naturally the bats that did not have wings died off while the bats with wings survived and continued to reproduce.
Will	At one point all Trumpet flowers had dull yellow flowers. Along the way, one Trumpet flower experienced a physical mutation that altered its DNA. That flower, now with an altered DNA, reproduced with a normal Dull Yellow Flower. The product of that reproduction was created with an altered DNA that caused it to change color, probably a pink or so. Over time, pollinators chose the pink flower because it is easier for their eyes to see. This resulted in the yellow Trumpet flower slowly dying off because it could not reproduce due to the pink flowers taking all of the pollinators attention. After all the yellow flowers were gone, the pink ones started to become brighter to attract more attention.

Layla proposes that the possible selective advantage of parachute-like seeds in dandelions may lie in their ability to travel away from competitors—an idea she introduces with “I think” to mark that the idea is both her own and perhaps tentative. Nikita hedges in a different way, noting that there “may have been a gradual change” in the environment and implying that she is not sure what this change is. Will’s response does not include obvious hedging but does include a novel idea that color change in flowers was driven by a shift in pollinator eyesight. Will’s questions about this idea only emerge later during the interview.

In interviews, all three students at first focused their attention on the correctness or completeness of their answers. Layla first talked through her explanation for the evolution of dandelion seeds as follows:

Interviewer: Can you talk me through your thinking in your answer there?

 

Layla: Um well (pause) Obviously like traits that like animals or plants um evolve are to adapt to, I guess, like their environment and survive better. [I: mm hm] So I guess I just like um explained how their parachute-like structures let them like um travel and disperse [I: mm hm] with like their seeds and whatever. And then (pause 2s) yeah that’s all I explained. It wasn’t really in depth.

In this response, Layla begins by emphasizing the connection between adaptations and differential survival, a core idea that she marks as “obvious,” perhaps to signal that she understands this part of her response to be correct. She then, more tentatively (“I guess”) introduces her ideas about the adaptive significance of the parachutes for dispersal. She ends by noting that her written answer was not “in depth.” Despite sharing her idea about dispersal, Layla’s seems to primarily be understanding this moment of the interview as about reporting knowledge that may be evaluated. Her use of the words evolve, adapt, and survive align with canonical explanations. When the interviewer follows up a few minutes later to ask if her explanation made sense to her, Layla answers, “I don’t know. I mean, I was- I was looking forward to getting this checked.” By this she seems to suggest that she would be more sure of the correctness of her answer if it had been evaluated by her instructor.

Evidence from the early parts of Nikita and Will’s interviews are similar. Each reflects on their written response, offering some evaluation of the quality of their answer. Nikita says, “Well I think- I mean- I guess it’s good enough. I don’t know. I just feel like I’m missing something,” and later recounts her concern with providing enough detail because, “on the test, I wasn’t specific enough.” Will begins by stating, “my reasoning sounds pretty solid,” but also “I’m noticing that I didn’t go into too much detail.” He further explains, as if to apologize, “I just know the basics of natural selection.” Such statements indicate that at the beginning of the interviews, students perceived the interview to be framed around *reporting knowledge* to the interviewer. Their self-evaluations seem intended to stave off criticism about the level of detail or signal uncertainty over whether their answer would be considered correct enough.

As the interviews progressed, the framings also shifted, making more room for discussions of students’ questions and tentative ideas. The interviewer began to explicitly prompt for what “didn’t make sense” and to invite students to imagine and speculate. All three students began to articulate questions and offer tentative explanations that they further developed by engaging in evolutionary reasoning. In the next section we elaborate on what this thinking looked like for each student, highlighting the posing of biological questions and generative thinking that emerged during this part of the interviews.

### What does student-driven sensemaking about natural selection look like?

In this section we present illustrative excerpts from the sensemaking we identified in our interviews of three focal students Layla, Nikita, and Will. For each student we describe one of the moments in which they identified and elaborated on a gap or contradiction—something that did not make sense. Then we describe the tentative ideas they proposed to fill that gap with a possible explanation. Our focus in presenting these examples is not to evaluate the correctness of these ideas, but rather to present them as examples of the kind of tentative reasoning that we see as the hallmark of scientific sensemaking. These excerpts also illustrate some of the discourse moves made by the interview helped initiate and sustain a sensemaking framing.

### Layla: It’s a little random to me

Following Layla’s reflections on her written response, the interviewer again asks if there is anything Layla “feels unsure about.” At first, Layla says that she “doesn’t know how they looked before”; That is, she couldn’t picture what an ancestor of a dandelion seed looked like. With encouragement to speculate, Layla proposes that perhaps the ancestral seed was “less fluffy” or, pointing to the picture, just the little seed hanging from the parachute. The interviewer follows up to ask Layla how the little seed could have evolved into a parachute. At first, Layla admits, “I just wrote a genetic mutation. . .I guess I don’t know what that means.” Here, Layla’s use of the term “genetic mutation” and apologetic tone over not knowing what it means again aligns most strongly with *reporting knowledge*. The interviewer follows up on Layla’s statement about genetic mutation:

Interviewer: But when you said mutation, you said I don’t know what that means. What part of it do you not know what it means?

Layla: Not th- like I- I just don’t know like how- how- how it happens

Interviewer: Yeah

Layla: It just yeah happens randomly [rising intonation]

Interviewer: Right [chuckles]

Layla: Yea and it happens to be something better [rising intonation] It’s just- it’s a little random to me

Layla’s skepticism over a random mutation being able “to be something better” appears to mark a more stable shift into a sensemaking framing for Layla. In her explanation, Layla had written that the origin of the trait had something to do with mutations, but she then expressed confusion over how a parachute could suddenly appear. This was a gap that did not make sense to her. To encourage Layla to think about this gap, the interviewer poses a follow-up question, and Layla responds by offering her ideas:

Interviewer: And you’re thinking about, and you’re saying like, you know, that like from what you’ve learned about natural selection, that a mutation can cause things, but you’re still not exactly sure like, how it can do that. Does it seem possible that there could be a mutation that would just like make a parachute?

Layla: I mean I don’t know (pause 2s) like again, I don’t know how it looked before, right? [I: right] so I don’t know how drastic of a change the parachute-like structure was…. If like it changed its structure completely maybe there were like multiple [rising intonation] umm evolutions like steps, right? Maybe this is just like the newest [rising intonation] version right?

Interviewer: Yea I mean maybe. So, if you imagine it started as just like a little regular seed can you imagine some steps in between?

Layla: (pause 5s) Mm I don’t really have a good imagination.

Interviewer: [short laugh] It’s ok

In the above, Layla expands on the problem she has identified but remarks that it is challenging to reason about evolution without knowledge of the ancestral state. Yet she begins to raise the idea that there may have been multiple steps. The interviewer attempts to encourage Layla to “imagine” the intermediate steps between a seed and a parachute, and at first Layla hesitates and says she does not have a good imagination. However, after a short pause Layla begins to talk aloud and, without further intervention from the interviewer, begins to construct a stepwise account of the evolution of a parachute-like seed from an initial seed through first a stick, then a softer bit, and eventually a full parachute (edited lightly for length and clarity):

Layla: And then maybe… at first the seeds would just be in the middle and they’d fall. And then maybe there was like a genetic mutation where it like stuck out a bit. Then it … it wouldn’t fall like down here it would fall a little bit further…. And then I guess, like the stick turns into like something that flows a little bit, like, like this, it’s softer. And then it can fly away a bit more and then the shape changes a little bit and then ultimately changes to a parachute-like structure. Yeah, that’s my evolution.

In this proposed explanation, each new form originates from a mutation, and each form allows the seed to travel further from the parent plant to avoid competition. Layla has begun to make sense of the emergence of complex structures that she had previously found challenging to imagine.

### Nikita: I don’t know why they don’t just crawl up a tree

Nikita, in her written response, chose to explain evolution of wings in bats, which she did by explaining that bats with wings must have had some “advantage” in the environment (Table 1). Her response hints at a gap by stating, “there may have been a gradual change in their environment.” When asked to talk through her answer in the interview, Nikita elaborates on this gap:

Nikita: I said there’s a bit of a gradual change in the environment where flying would be an advantage. But I just don’t know. The hard part for me is thinking about what it would be that would be the change. I just said something happened, but I don’t know what. I mean, it could have been a predator, the climate, I don’t know how to specify. Because I know on the test, I wasn’t specific enough… [elaborates on her exam answer]. I think the professor said that wasn’t specific enough. Yeah, sorry, I went off.

 

Interviewer: No, no, that’s OK. I mean, so do you feel- well, let’s just keep going with what makes sense to you.

After elaborating on what she is unsure about, Nikita shifts to concerns about the specificity of her answer, a statement that could shift the interview into a discussion about what Nikita does and does not know. The interviewer responds by encouraging Nikita to “keep going with what makes sense to you” and then by explicitly by asking, “Is there anything about that story about the bat that actually doesn’t make sense to you?” Nikita responds by saying, “I don’t know. I feel like maybe it’s just like it feels like the wings just came out of nowhere.” Over the next few minutes, Nikita begins to shift into a framing that is more in line with sensemaking as she tries to address the gap that she was bothered by. She pauses, and then says:

Nikita: Like let’s say it was, I don’t know, let’s say it was like a predator who was on the ground. So, they wanted to have wings. So, it was beneficial to have wings. Yeah, the bats who didn’t have wings would get eaten. And the bats who did would. Yeah. That’s how I think of it.

Nikita’s use of the phrase “let’s say” and “I don’t know” conveys that she is proposing tentative ideas. She is examining whether predation could possibly explain why wings might have been beneficial. “That’s how I think of it” could indicate that Nikita is aligning herself with a sensemaking framing. The evidence for sensemaking is even stronger when Nikita, like Layla, starts articulating what is still confusing to her. Nikita had expressed skepticism that “wings just came out of nowhere” and over the course of several minutes proposes a series of transitional forms featuring a “webbed” paw that could help her explain the evolution of a mouse-like creature into a bat. Even with this tentative explanation Nikita still expresses dissatisfaction with her explanation. She returns to the question of the ecological context in which wings would be an advantage, this time articulating a gap in the logic of evolving wings to escape a predator:

Nikita: But I don’t know why they wouldn’t just like crawl up a tree…. Yeah, I mean, I guess the predator could evolve to crawl up too, I guess. But then it can also have wings.

While Nikita understood predators to be a valid answer initially, she now articulates a problem with this explanation: Why evolve a trait as complex as flying when crawling would also provide a means of escape? Even more puzzling is the problem that predators could evolve too, meaning that nothing stops them from evolving to crawl or fly after prey. In her sensemaking, Nikita is raising questions about the ecological context of evolution as well as ideas about the possibility of a co-evolutionary arm’s race. Nikita’s posing of a question, her tentative explanation, during which she says “I guess” two times, and her identification of a further complication (the predator could also evolve wings) all suggest that Nikita is actively working through these ideas in the moment.

### Will: Why would they switch to the pink one?

Will’s written explanation for the evolution of a color change in trumpet flowers included expected core ideas of genetic variation, differential selection, and inheritance (Table 1). In the interview, Will summarizes these ideas fluidly but also hints at a potential gap in this explanation by using the hedging phrase “must have been.”

Will: Because a random genetic mutation, just from there being so many variations of genetic material, just happened to cause one to be pink just by nature, and then pollinators must have been attracted to that one specifically, which allowed the pollinators to spread that to other areas and make the pink one more popular.

The interviewer, revoices and amplifies this uncertainty by stating, “the pollinator has some preference for some reason.” Following this restatement Will elaborates further on this gap:

Will: Why would they [switch from yellow to pink]? Because, I mean, no, no, no. Well (pause 2s) yeah, that’s what I was trying to get at with the anthocyanins that we had in another question. It was kind of like this but with a little more detail. Because I was trying to think, if the pollinators were pollinating the yellow one already, that means they can already see it and they can already pollinate it. [I: right] Why would they switch to the pink one? So, this is basically just what I was told. This isn’t even an actual in-depth analysis of what’s happening.

 

Interviewer: Yeah, that’s kind of what I’m curious about.

 

Will: I’m trying to process it right now.

 

Interviewer: I’m less interested in what you were told and more interested in, like, what do you actually think about it? Yeah, so you’re saying-

 

Will: One idea right now is that some pollinators can see the yellow one and then maybe the majority of all pollinators can see the pink one. And- but then there would still be those pollinators going to the yellow one, so why would it go extinct is what I’m trying to figure out? Because that would mean that something would have to be altered with the pollinator probably (pause 2s) that would make it prefer the pink one.

In line with a sensemaking framing, Will is both posing and trying to answer his own questions during this part of the interview. He is sharing ideas as they come up for him and asking himself questions about whether those ideas satisfy his own questioning. Specifically, Will asks what would motivate a shift in host plants if the pollinator already had a relationship with the yellow flowers, and whether a shift in hosts could cause the yellow flowers to go extinct. With these questions, Will’s sensemaking continues, leading him to reason about the mechanisms and consequences of coevolution and host shifting. This framing was likely encouraged by the interviewer stating that she was “curious” and interested in Will’s thinking.

### Students’ reflections on sensemaking

In this section we return to the statements we used to introduce this paper. Each student made statements that highlight the tensions between the sensemaking that they did in the interviews and what they typically understand to be valued in their biology courses.

Layla spent 45 minutes discussing the evolution of bat wings and color change in flowers. As she and the interviewer wrap up a discussion of the possible molecular changes that could cause color changes in flowers, Layla spontaneously shifts to discussing her biology class:

Layla: Yea. And like I often find myself like being really confused like this especially in Bio [I: mm hm] And then I’ll like try to think about it like and then I do a practice quiz or a practice test or whatever, and then we don’t get that deep in like the questions but then- then I feel like I wouldn’t truly understand any of it unless I know the answer to this like really specific or maybe basic fundamental like concept. Like I feel like I need to build off of these concepts to understand everything else. But, if I keep thinking about it and they don’t even ask me about it, it’s like maybe I should just memorize- like what’s quizzed.

Layla describes a challenge she faces as a biology student when she encounters something confusing. Although she tries to take practice tests, she notes that the thinking often doesn’t “get that deep.” Layla seems to see the value in genuinely understanding fundamental concepts. At the same time, she reflects that efforts to “keep thinking” are not rewarded; it might be a better strategy to “just memorize.” Ultimately, Layla appears uncertain whether the kind of thinking prompted by the interview will serve her in her biology class.

Nikita raises a similar issue at the end of her interview:

Nikita: Like the test won’t ask me about the part that I’m confused about. [I: It won’t?] Yeah, which is why I just ignore it [laughs]

 

Interviewer: Right, I see. So, what does the test want you to say?

 

Nikita: It asks, explain how the trait, the new trait evolved from ancestors that did not have the trait. And I just- like that’s why I said I needed to be more specific, because I gave the broad answer instead of thinking about, like, this part.

 

Interviewer: But you also said that part is, like, not important for the test.

 

Nikita: Yeah, because I feel like the broad answer is good enough. [I: Yeah, yeah.] Oh, something happened - don’t know what.

Nikita’s interview had mostly focused on what she felt confused about—both the ecological rationale for wingedness and the evolutionary steps that would benefit intermediate forms. Like Layla, Nikita spontaneously remarks that the test won’t ask her about the things she is confused about. When the interviewer asks what the test is looking for Nikita initially says that she needs to include specifics, perhaps like those she and the interviewer had been working through together. She then shifts, acknowledging that often a “broad” answer is good enough. What Nikita seems to be saying is that a good exam answer need only state that the bats evolved wings by natural selection, not to provide an ecological rationale. Such an answer, she adds in a dismissive tone, only says that “something happened- don’t know what.” Like Layla, Nikita does not seem to see the sensemaking she has engaged in during the interview as something that is typically valued on an exam.

Will also spontaneously reflects on his sensemaking about both the flower and bat examples, saying:

Will: I think (pause 2s) I think (pause 2s) focusing on the right answer limits my brainstorming you know what I mean? That’s why I was- I originally chose what I know was right. I know that bats evolved from whales, and some- I know that fins are similar, and they have common ancestors. So, I knew that was right in my mind, so that’s what I started with.

Will describes that he started the interview by sticking with what he knows to be right. For example, he has learned before about the link between whale and bat limbs. However, Will also acknowledges that this orientation limited his brainstorming, and describes how this shifted as the interview went on:

Will: I didn’t want to start with something I wasn’t sure I was right about. I’m not sure about the sticky [feet] thing^1^, but as you got me more comfortable, and you were, like, basically alluding to the fact that you’re looking for my explanation, not my answer. Like, that’s when I got more comfortable and open-minded, and I was looking at subjects that I actually think, rather than regurgitating things that I’ve been told. Because there’s nothing relying on- Like, there’s no right or wrong answer. You’re just looking for my explanation. And that’s why I had a little trouble with like the [in-class writing assignment] because I was looking for- I’m like, this is a question, there must be a right answer.

Will’s elaboration contrasts his experience in the interview with his approach to answering the in-class question. In the interview, Will came to understand that the interviewer was looking for his ideas, not just an answer. Like both Layla and Nikita, Will is describing how classroom contexts reinforce framings of reporting on what is known rather than making sense of what is confusing or puzzling.

#### What can we learn from these cases?

We have illustrated that community college students in a first-semester biology course raised substantive evolutionary questions and reasoned about possible explanations when they saw that their questions and ideas were valued in interviews. Layla questioned the process by which a random mutation could lead to the evolution of a complex trait and proposed a sequence of intermediate forms that could bridge the gap between small seeds and parachute-like seeds. Nikita wondered both about the series of steps linking a mouse-like ancestor to a winged bat and about the ecological context of wing evolution, raising questions about the advantage of flying and proposing, without using the formal term, the idea of an evolutionary arm’s race. Will questioned the mechanisms of pollinator host switching, wondering both what would motivate a pollinator to switch hosts and what sequence of co-evolved changes would make such a switch possible. Their questions and ideas went beyond answering how a trait evolves by natural selection to discussing questions that have motivated scientists working in the field of evolutionary biology: How do complex traits evolve? What is the relationship between ecological context and evolution? Why do well-adapted traits change? What are the consequences of coevolution? Not only are these questions of interest to the field, but they also seemed to engage students. When talk turned to what didn’t make sense, students became more animated, expressing confusion and dissatisfaction coupled with a desire to know more. With encouragement, students shifted from worrying about grades to authoring their own mechanisms, often punctuating these explanations with markers of pride as in Layla’s declaration, “That’s my evolution.”

These three students had no special prior experience or coursework. And the patterns we describe in these focal interviews are typical of what we have seen across the larger set of interviews (N = 28) conducted with first and second-year students at two institutions: one community college (N = 16) and one private university in the Boston area (N = 12). While our sample is small, we nevertheless suspect that the ability to sense-make about evolutionary phenomena may be an asset that many students bring to biology classrooms.

Evidence of students’ ideas and questions was evident even in students’ written responses (Table 1), but became much more elaborated in the interviews, revealing confusions that students were eager to think and talk about. Students’ engagement with sensemaking was likely supported, as Will noticed, by the interviewer’s attempts to ask students about their own explanations, not answers. In line with prior work, students seemed to perceive a shift in the epistemological framing of the interview (Russ et al. 2012). Early on students raised concerns about their answers noting the lack of detail and referencing missed points on exams. This expectation limited their willingness to brainstorm or voice their confusions. As the interview continued, the interviewer repeatedly asked them about what did and did not make sense to them and encouraged them to engage in speculation and imagination. These moves seemed to help signal to students that this interview was a space in which they could raise their own confusions and offered tentative ideas.

Interestingly, all three interviews also ended with students spontaneously returning to their perceptions of the lack of value and opportunities to think deeply in their biology course. Layla, who had over the course of the interview drawn out her “webbing theory” of bat evolution expressed that she wanted to show it to her professor, but then worried that he might tell her it was incorrect. Nikita confessed that she would not feel comfortable sharing this kind of thinking on a test and would be more likely to “stick to the broad answer.” Will expressed that he still wasn’t satisfied and wanted to continue thinking about these ideas but also acknowledged that in class he isn’t supposed to think about all these extraneous ideas: “I’m just supposed to know that I think I’m just supposed to know the basics that they did evolve.”

This too is a common refrain across the interview set and may help explain why biology students consider falling back on memorizing or giving expected answers rather than sharing their more in-depth questions or tentative ideas (Google et al. 2023). That students experience biology classes in these ways suggests a need to shift the epistemological framings of these learning environments.

### Implications for biology education

In this work we argue that students have questions about biology that they want to make sense of and ideas that they can begin to make sense with. We saw these questions and ideas arise in one-on-one interviews with a researcher who was attempting to intentionally support students’ sensemaking. In this section we consider implications for instructors and curriculum designers who want to create opportunities for sensemaking in classrooms. Despite the differences between interviews and classrooms, the patterns observed in these interviews align with and reinforce established ideas about sensemaking in science education scholarship.

One strand of work focuses on curricula and the value of intentionally designing in opportunities for students to encounter and raise questions about which they are puzzled (Manz 2015). As we noted in the introduction, some biology curricula have been designed to set students up to notice gaps and inconsistencies in observations or data about the biological world (e.g., Bierema et al. 2017; Bolger et al. 2021; Adams & Barth-Cohen 2024). Our interviews suggest the possibility that students themselves may have questions that could become the focus of classroom inquiry (see also Odden & Russ 2019b; Phillips et al. 2018), raising the question of how instructors might create opportunities for students to articulate and try to make sense of the questions they have—the things that don’t yet make sense to them. Taking these questions seriously could both deepen students' learning and be more engaging.

The framing dynamics of the interviews reinforce that approaches intended to center sensemaking require attention to epistemological framing so that students understand that instructors are genuinely interested in their questions and ideas, not just in what they know to be correct (Hutchison & Hammer 2010; Ginath & Southerland 2018; Svoboda et al. 2025; Heinrich et al. 2026). Such messaging can be communicated to students through both instruction and curriculum design (Russ 2018). In terms of instruction, “responsive teaching” approaches support student sensemaking by intentionally eliciting students' questions and ideas and incorporating them into instruction (Robertson et al. 2015; Watkins & Manz 2022; Svoboda et al. 2025). Teaching responsively challenges instructors to allow incorrect ideas to be part of the discussion rather than rushing to correct them. This is expected and appropriate—scientists, too, propose incorrect ideas as a natural part of sensemaking, and that process is understood to be generative rather than problematic. In practice, students' confusions only tend to surface once they feel safe proposing tentative ideas; if those ideas are met with immediate judgment, students are likely to retreat to saying what they believe will sound correct and stop thinking generatively. If, instead, space is made for students to raise and sit with the things that puzzle them, they can begin to make genuine progress in understanding the biological world.

Even so, responsive instruction alone cannot fully support sensemaking without accompanying shifts in curriculum and assessment that signal to students what kinds of knowledge and thinking are valued (Elby 1999; Russ 2018). Research on epistemological messaging in introductory STEM courses shows how syllabi, exams, and answer keys reinforce a framing of learning as the reproduction of known information for an authority (e.g., Heinrich et al. 2026). What our interviews suggest, however, is that students' capacity for sensemaking already exists, if perhaps beneath the surface. Students surfaced their questions and ideas even in written responses. In interviews, when students had the opportunity to raise what puzzled them and the time to sit with their questions without immediate evaluation, they did. The challenge, then, is to build learning environments in biology and across STEM disciplines in which students understand that making sense of their own questions and confusions is genuinely valued.

## Data Availability

Data available upon request from corresponding author

## Footnotes

1 In another part of Will’s interview, he, like Layla and Nikita, reasons through a series of transitional forms that could possibly explain the evolution of bat wings. Will’s idea involves the evolution of “sticky feet” that allow ancestral rodents to climb, proposing that wings would then evolve to address the harm of falling from heights.
